# Colloidal crystals by electrospraying polystyrene nanofluids

**DOI:** 10.1186/1556-276X-8-26

**Published:** 2013-01-12

**Authors:** Arnau Coll, Sandra Bermejo, David Hernández, Luis Castañer

**Affiliations:** 1 MNT, Electronic Engineering Department, Universitat Politècnica de Catalunya, Jordi Girona 1-3, Barcelona, 08034, Spain

**Keywords:** Electrospray, Nanospheres, Colloidal crystal, Multilayer, Metamaterial

## Abstract

This work introduces the electrospray technique as a suitable option to fabricate large-scale colloidal nanostructures, including colloidal crystals, in just a few minutes. It is shown that by changing the deposition conditions, different metamaterials can be fabricated: from scattered monolayers of polystyrene nanospheres to self-assembled three-dimensional ordered nanolayers having colloidal crystal properties. The electrospray technique overcomes the main problems encountered by top-down fabrication approaches, largely simplifying the experimental setup. Polystyrene nanospheres, with 360-nm diameter, were typically electrosprayed using off-the-shelf nanofluids. Several parameters of the setup and deposition conditions were explored, namely the distance between electrodes, nanofluid conductivity, applied voltage, and deposition rate. Layers thicker than 20 μm and area of 1 cm^2^ were typically produced, showing several domains of tens of microns wide with dislocations in between, but no cracks. The applied voltage was in the range of 10 kV, and the conductivity of the colloidal solution was in the range of 3 to 4 mS. Besides the morphology of the layers, the quality was also assessed by means of optical reflectance measurements showing an 80% reflectivity peak in the vicinity of 950-nm wavelength.

## Background

Self-assembly is a technological process resulting in an ordered structure of individual units without direct human intervention. Most often, this is the simplest technique to produce nanoscale structures, and this is the main reason of the recent wide interest, as revealed by comprehensive compilations. Some reviews [1-4] exhaustively describe the different existing technologies, mainly based on electrophoretic forces [5], capillary forces [6,7], dip coating [8,9], and ink-jet printing [10], among others.

Top-down approaches, such as lithography or ion sputtering, have smaller chances to be able to produce large-scale low cost materials than bottom-up wet methods, despite the limitations of techniques such as spinning or sedimentation. Mono- and multilayers of nanospheres have a huge number of promising electrical and optical applications [11-14]; some benefiting from the high surface-to-volume ratio to, for example, foster a new generation of ultrafast bulk battery electrodes [15], scaffolds of macroporous materials [16,17], while others benefit from the dimension of the periodicity of three-dimensional (3D) structures making them suitable for photonic [18-20] or terahertz applications [21].

The technique used in this work is known as electrospray. It consists of producing a fine aerosol by dispersion of a liquid by application of a high electric field between an emitter, usually a thin needle, and a flat electrode. Above a given voltage threshold, a Taylor cone develops [22] and the liquid tip becomes unstable breaking into small droplets. The main application of electrospray is found in the ion source of mass spectrometers, although it has also been recently used as a nanoparticle deposition method [23-25], polymer thin film deposition [26], or to create photonic balls [27]. To our knowledge, electrospraying of nanofluids or colloidal solutions of nanometer-size spheres to produce full 3D self-assembled crystals has not been reported so far. A very comprehensive work on state-of-the-art colloidal crystals has recently been published [1] where a few indicators of the crystal quality produced by the various techniques are summarized and compared, namely the thickness, area, deposition time, and optical quality.

**Figure 1:**
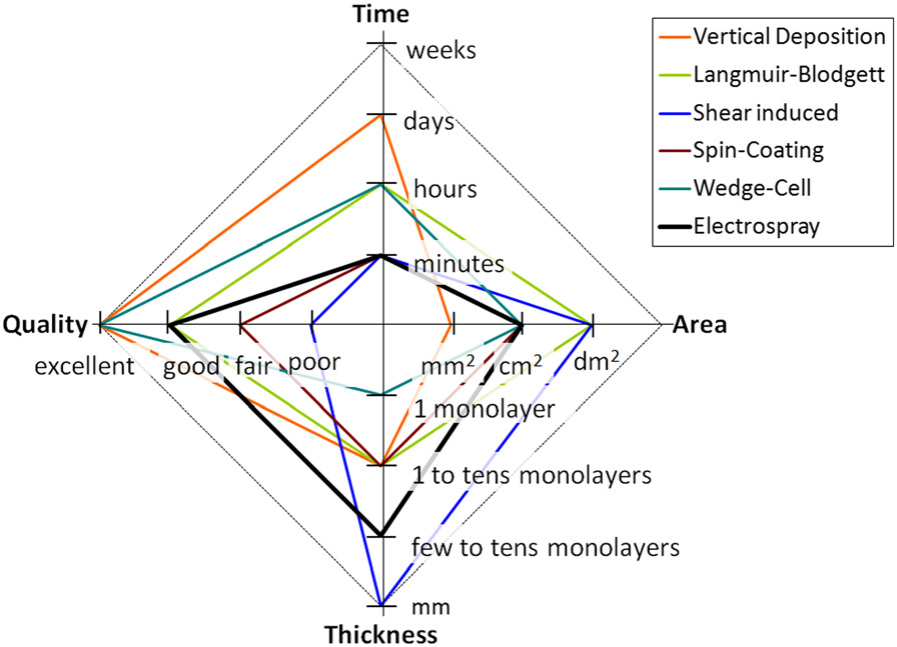
**Radial plot of quality indicators for some of the most relevant colloidal crystal fabrication techniques.** Deposition time, area, thickness, and quality of the photonic crystal are compared. The technology introduced in this work is the electrospray, in solid black.

We have drawn in Figure 1 a radial plot of selected information from Table one in [1] for some of the deposition techniques reported there. We have not included the indicators concerning four techniques, namely motor-drawing, sedimentation, cell confinement, and air-water interface due to the poor results compared to the rest.

As can be seen in Figure 1, the most successful techniques exhibit good to poor optical quality, but deposition times are long and the crystal size is in the range of square millimeter of area. It can be seen that the only technique being able to provide wafer-size colloidal crystals (tens of square centimeter in area) in some minutes is the spin-coating technique. It can be seen from this plot that the combination of large area, tens of monolayers of thickness, range of minutes to fabricate, good or excellent optical quality of the crystals, and 3D order is difficult to achieve in most of the techniques. In Figure 1, we have highlighted the results that we have achieved with the technique we are describing in this paper: the electrospray. Using this technique, we were able to deposit up to tens of monolayers, in a few minutes, in square centimeter size, with 3D order, and with good quality. These remarkable results, which are described in the sections below, compare quite well with the other state-of-the-art techniques reported in Figure 1. Thus, we can claim to have achieved a good compromise between large area and low deposition time, achieving good quality of the colloidal nanostructures.

In this work, the deposition conditions, such as flow rate, solution concentration, electrical potential, and distance between electrodes, are examined to find the optimal deposition conditions to create 3D self-assembly crystals. In the electrospraying deposition of particles on a substrate, several forces and physical phenomena are involved. In the short range, electrostatic forces are important, in addition to surface tension and capillarity, to explain particle adhesion to surfaces and particle chain, formation, or self-assembly. Coulombic and multipolar dielectrophoretic forces contribute to the total force acting on the particles, thereby affecting the adhesion regimes. The sign and magnitude of the dielectrophoretic component depends on the Claussius-Mossotty factor [28], which depends on the values of the permittivity of the particle and of the medium. In this work, we have observed a set of experimental conditions leading to net attractive forces between particles, so they aggregate in the three dimensions of the layer growth. Scanning electron microscope (SEM) images and optical measurements are also shown to demonstrate the quality of the fabricated colloidal crystals.

## Methods

**Figure 2:**
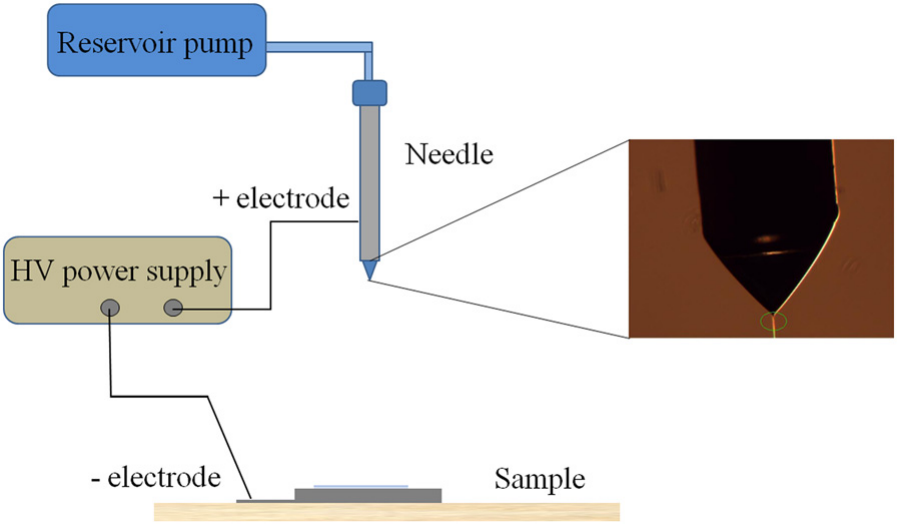
**The electrospray setup.** Schematic view of the experimental setup and an enlarged image of the tip of the needle with a Taylor cone and a jet of 4 μm circled in green.

The electrospray setup consisted of an infusion pump from B. Braun SA (Melsungen, Germany), an OMNIFIX (Braun) 5-ml syringe, a Hamilton needle (600-μm outer and 130-μm inner diameter; Hamilton, Bonaduz, GR, Switzerland), and an Ultravolt high-voltage bipolar source, −15 kV to +15 kV (Ultravolt, Ronkonkoma, NY, USA). The deposition area was placed inside a glove box with controlled N_2_ atmosphere. Figure 2 shows schematically the experimental setup.

In this work, silicon and glass substrates were both used, and they were covered either with aluminum or ITO patterned layers typically 120-nm thick. ITO electrodes allow optical observation as it has good optical transmission [29]. Polystyrene nanospheres, 360 nm in diameter, were electrosprayed targeting these patterned electrode areas. The main parameters that were explored in the experiments were the value of applied voltage, the distance from the needle to the substrate, the solution concentration, the solution conductivity, and the deposition time.

The first efforts were devoted to finding suitable experimental conditions to get a stable Taylor cone at the tip of the needle. This involved changing the distance from the needle to the substrate and changing the bias conditions. We found that a Taylor cone was created when the distance was typically between 10 to 15 cm and the applied voltage difference was between 7,500 and 14,000 V. Differences in the deposition results were also found when the substrate was grounded rather than negatively biased. Our best results were obtained when −1,000 V was applied to the substrate and +9,000 V was applied to the needle.

Once the conditions for Taylor cone creation were found, the effects of the solution pumping rate, solution concentration, and solution conductivity were explored. No effects on the order of the deposited layers were found by just changing the solution concentration. Our best results were found for 350-μS solution conductivity and 2.2-ml/h pumping rate, provided the voltage conditions were as described above, +9,000 V at the needle and −1,000 V at the substrate. For these conditions, the deposited film was composed of tens of ordered layers. Additionally, increasing the conductivity to the range of 4 mS by adding formic acid to the solution and decreasing the concentration of nanospheres tend to produce smaller droplets and layers of scattered nanospheres.

In our experience, to get ordered layers, some liquid of the aerosol is required at the surface of the substrate and, once the conditions to get a Taylor cone are satisfied, only the pumping rate and the solution conductivity seem to play an important role and not the solution concentration. A summary of some of the experimental conditions we have explored is shown in Table 1. Only the conditions leading to a Taylor cone formation are shown.

**Table 1:** List of the most relevant experimental conditions in the electrospray deposition of 360-nm polystyrene nanospheres

**Distance (cm)**	**Needle's voltage (V)**	**Sample's voltage (V)**	**Deposition rate (ml/h)**	**Conductivity (μS)**	**Dissolution**	**Qualitative assessment**
10	10,000	−2,350	0.41	8.35	50:50 isopropanol/water nanopolystyrene	Few dispersed nanospheres
10	7,500	−2,500	0.74	8.35	50:50 isopropanol/water nanopolystyrene	Few dispersed nanospheres
10	14,000	0	1.3	350	Water nanopolystyrene	Few dispersed nanospheres
14	14,000	0	0.3	350	Water nanopolystyrene	Few dispersed nanospheres
14,5	11,570	0	2	350	Water nanopolystyrene	Lots of dispersed nanospheres
14,5	9,000	−1,000	0.2	350	Water nanopolystyrene	Few dispersed nanospheres
14,5	9,000	−1,000	0.2	350	Water nanopolystyrene	Few dispersed nanospheres
14	9,000	−1,000	0.6	4,100	50:50 water nanopolystyrene/distilled water + 1.5% formic acid	Semi-covered layer of scattered nanospheres
14	9,000	−1,000	2.2	350	Water nanopolystyrene	Tens of 3D ordered layers

In all the processes, the humidity was monitored during deposition and typically was 20%.

## Results and discussion

**Figure 3:**
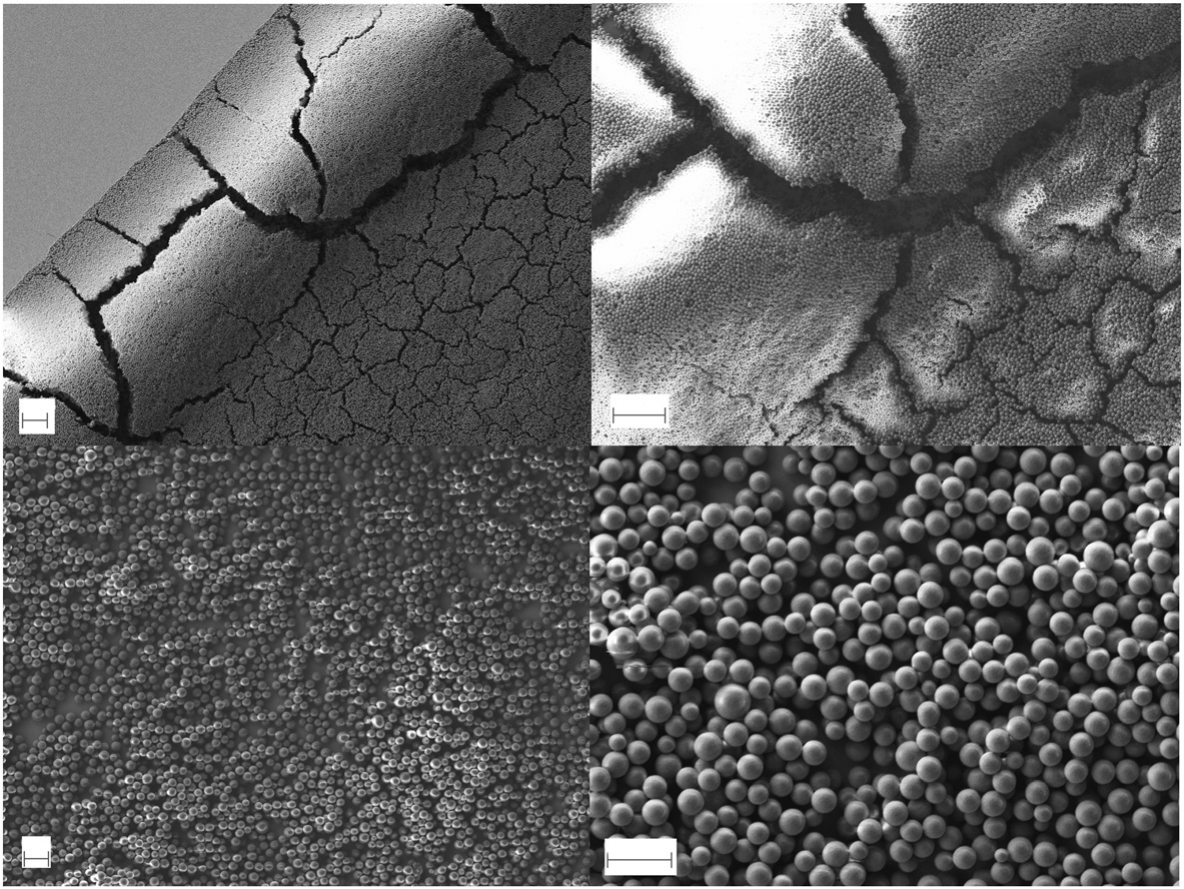
**SEM pictures showing a layer of 360-nm-diameter nanospheres after droplets falling onto the substrate dried.** In the top images, the scale bar is 10 μm, and in the bottom images, it is 2 μm.

**Figure 4:**
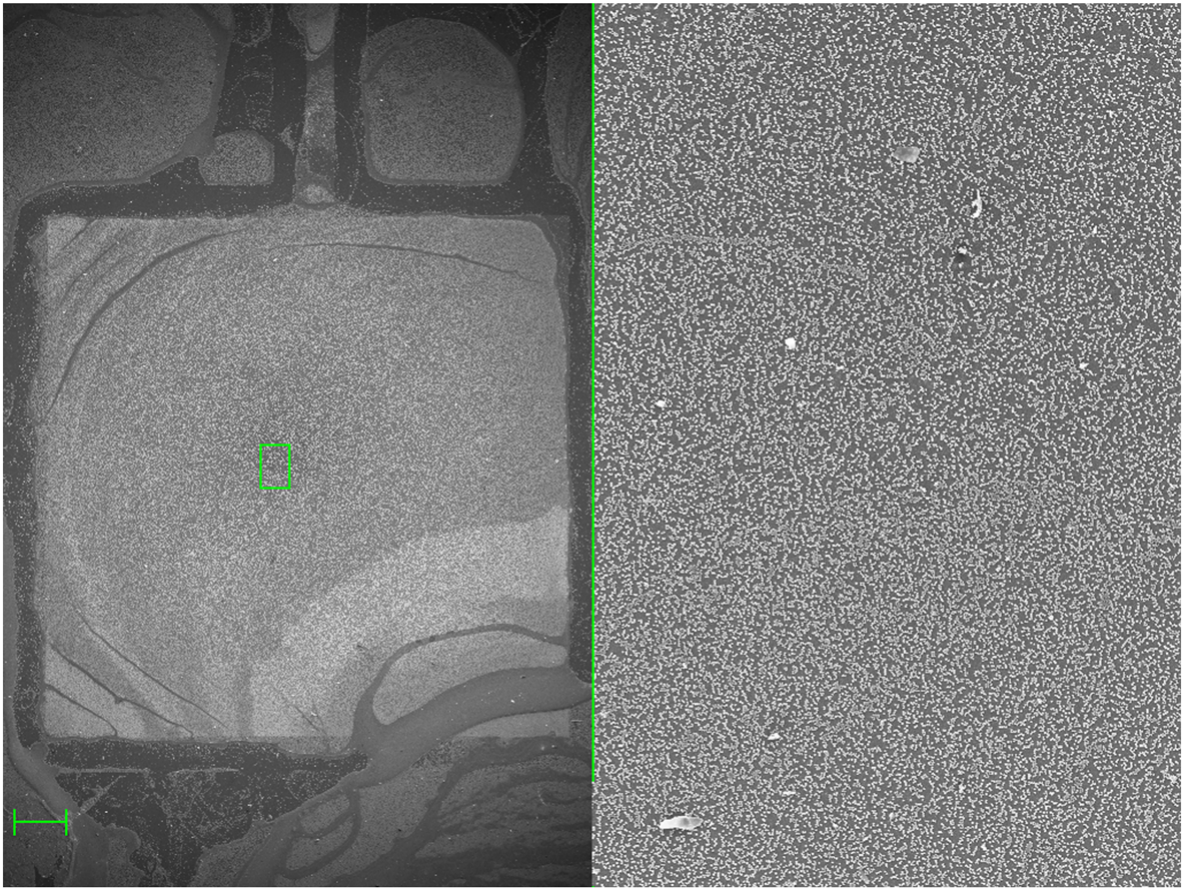
**Semi-covered layer of scattered nanospheres.** SEM pictures showing a monolayer of 360-nm polystyrene nanospheres deposited under the conditions shown in the eighth row of Table 1. The semi-covered monolayer follows the patterned contact, a squared electrode in the center of the left image and a path for electrical conduction at the top. Scale bar is 200 μm.

**Figure 5:**
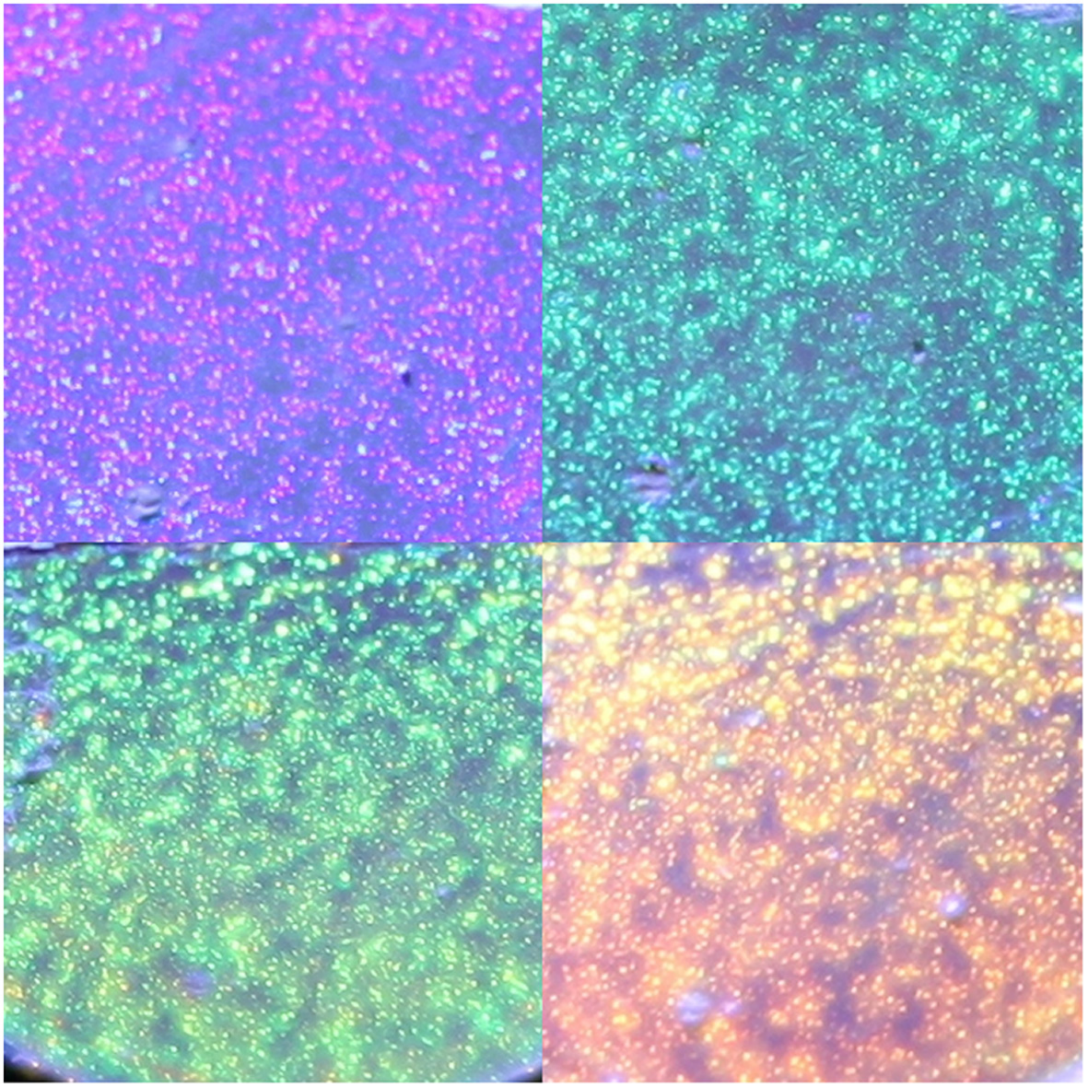
**Front surface view of an electrosprayed layer.** Light is coming from four different incident angles at 55°, 35°, 30°, and 20°, from top left to down right, and reflecting light corresponding to purple, blue, green, and orange wavelength. The sample displayed area is 5 × 5 mm^2^.

**Figure 6:**
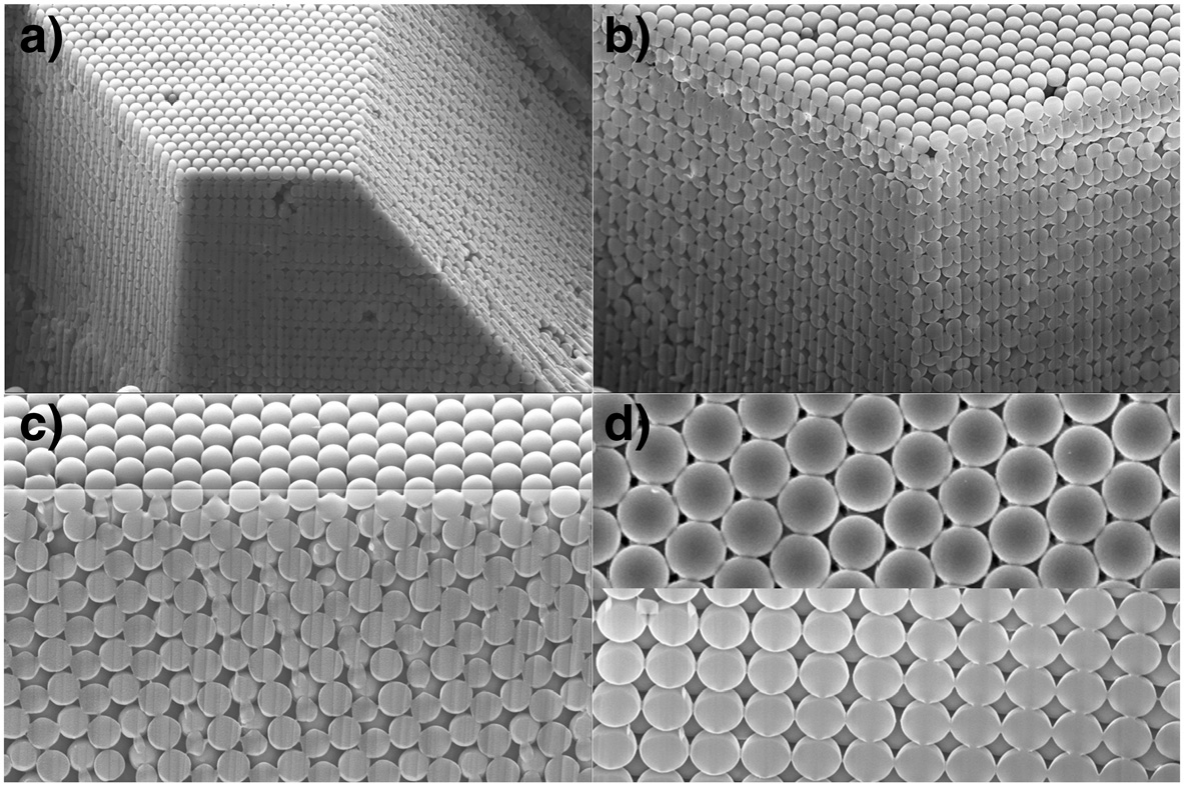
**SEM pictures of 360-nm-diameter polystyrene nanosphere layers.** (**a**) Cut surface showing [1 0 0] and [1 1 1] ordered facets, (**b**) close view of the perpendicular cut, (**c**) close view of the [1 1 1] face, and (**d**) top view of the [1 0 0] (top) and [1 1 0] order (bottom).

**Figure 7:**
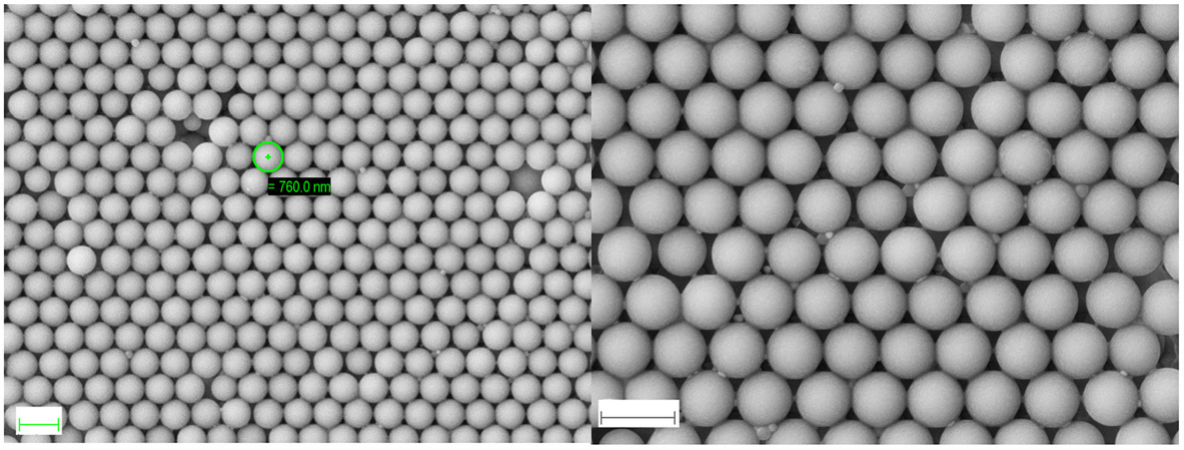
**SEM pictures of 760-nm-diameter polystyrene layers.** Scale bars are 1 μm.

**Figure 8:**
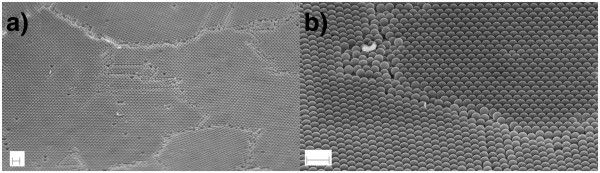
**Top view of large domains of polystyrene nanosphere layer.** SEM pictures of a colloidal crystal of 360-nm-diameter polystyrene nanospheres electrosprayed onto a silicon substrate deposited under the conditions described for Figure 6: (**a**) surface of the crystal showing the several domains and (**b**) a closer view of the dislocation between domains. Scale bars are 1 μm.

Following the experiments shown in Table 1, in this section, SEM observations and optical measurements are shown. When the conditions for a Taylor cone formation are not met, drops fall on top of the substrate, and when they dry, no significant order is observed in the nanosphere aggregation, as can be seen in Figure 3. The results obtained using the experimental conditions described in Table 1 can be summarized into two main groups: (1) some order is reached in semi-covered areas (Figure 4), and (2) complete 3D order is achieved in the whole area (Figures 5, 6, 7, 8).

Figure 4 shows the SEM pictures of a layer deposited using the conditions reported in the eighth row of Table 1. As can be seen, the layer involves scattered nanospheres with no 3D order. Metal areas are patterned on the surface of the substrate to define electrode areas that, when high voltage is applied, act as collection points where the nanospheres are self-assembled.

A number of observations were made on the films deposited for the conditions in the last row of Table 1. We first took optical pictures of the front surface when illuminated by a warm white LED (3,000 to 3,500 K) light at different incidence angles. This is shown in Figure 5, where the iridescence of the material can be seen.

Surface and in-depth SEM observations have also been performed, and the results are shown in Figure 6. Figure 6a shows a side view of the deposited layer after we performed a focused ion beam (FIB) milling. A closer view of the orthogonal corner in Figure 6a is shown in Figure 6b, where the (100) order of the top surface and of the two orthogonal planes etched by the FIB can be seen. A closer view of the edge of the top surface and of the inclined plane can be seen in Figure 6c, where the (100) and (111) orders are clearly seen. This is further seen in Figure 6d, where the (110) and (100) faces are also shown. The results shown in Figure 6 clearly demonstrate that the order of the self-assembly extends tens of layers in depth, reaching thicknesses of more than 20 μm, although we have not found a fundamental reason to prevent the formation of thicker layers with similar order, provided the deposition time is increased.

Polystyrene nanospheres of 760-nm diameter have also been deposited, reaching 3D ordered structures as well. Figure 7 shows 760-nm-diameter polystyrene nanospheres deposited under the same conditions shown in Figure 6: +9 kV needle bias and −1 kV substrate bias. The dissolution was an off-the-shelf distilled water solution of 760-nm polystyrene nanospheres, the pumping rate was 2.2 ml/h, and the deposition time was 10 min.

A macroscopic observation of the surface of the deposited layers demonstrates the existence of several domains of tens of microns wide. Inside every domain, the same order is kept, and dislocations can be seen in the frontiers between domains, as shown in Figure 8. Less than 0.5% defects in average are found inside each domain.

The experimental arrangement involves a very high voltage between a sharp electrode above a larger and flat electrode. It is well known that this arrangement creates an electric field distribution involving large gradients. This is the origin of the dielectrophoretic force that the nanospheres are subjected to. From our observations, we have first witnessed that below a certain value of applied voltage for a given electrodes distance, no 3D ordered layer is deposited, and this may be consistent with the threshold electric field value for Taylor cone formation and that postulated by Schwan and Sher [30] for chain formation, thereby indicating that neither conditions for aerosol formation nor particle aggregation are satisfied. We have also seen that our best results are obtained when a moderate value of the solution conductivity is used and when some liquid from the aerosol reaches the substrate. This may be consistent with the value and sign of the Clausius-Mossotty factor to create predominantly attractive forces between particles that tend to aggregate. Moreover, we have observed that when the conductivity of the solution is much increased, the layer deposited consisted of predominantly scattered nanospheres, which is consistent with dominant repulsive forces between them as a result of particle dipole interaction [31] and the dielectrophoretic force from the main field created by the needle tip and the bottom electrode.

The detailed explanation on how the dielectrophoretic force on the particles stimulates the orderly deposition of the spheres requires further work as particle alignment and enhancement of net electrostatic adhesion force have been described for xerographic applications [31]. Our work opens the way to investigate other electrode geometries and spatial and temporal dependence of the electric field to further improve deposition.

**Figure 9:**
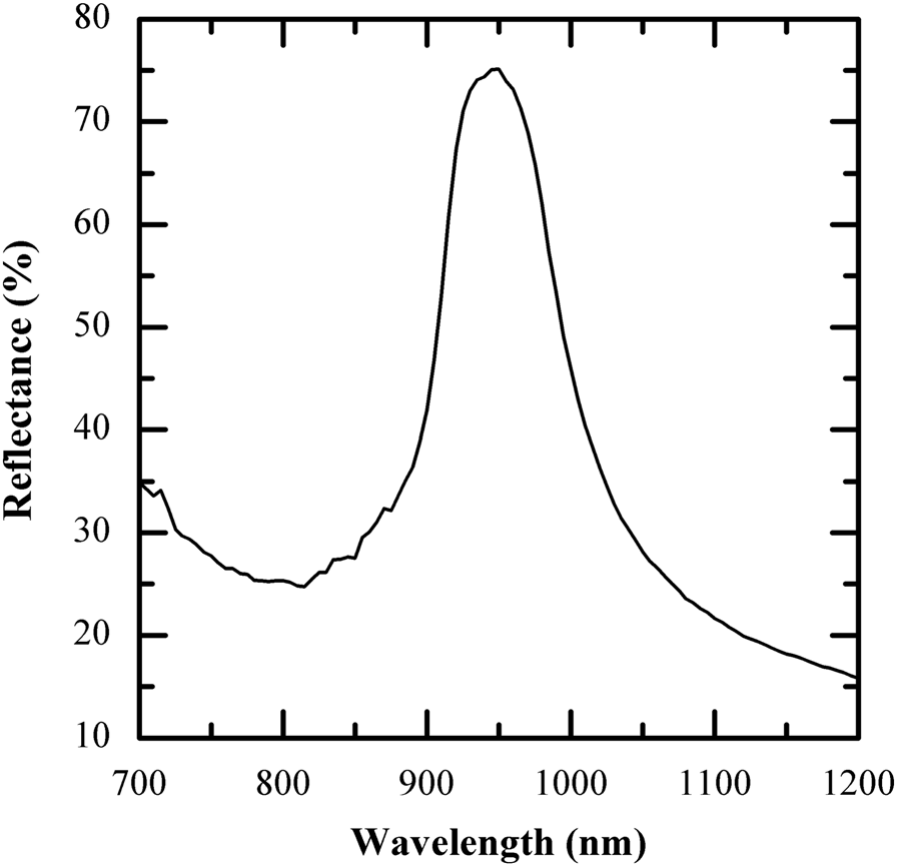
Reflectance measurements of a 360-nm-diameter nanosphere layer.

To complete the study on ordering quality, we have measured the optical reflectance of the crystals by infrared spectroscopy (see Figure 9). Spheres pack on an ordered combination of face-centered cube (FCC) and hexagonal close-packed (HCP) lattice, as observed from SEM images (Figure 6). The photonic band structure of an FCC lattice of dielectric nanospheres does not allow the opening of a full optical bandgap, neither HCP arrangement, but only of a pseudo optical bandgap along the L direction of the Brillouin zone [32]. As expected, there is a reflectivity increase in the spectral region 929 to 980 nm peaking close to 950 nm, with a maximum value of 75.1%. This result is consistent with theoretical calculations carried out using the plane wave expansion method, which predicts a relative stop band in the normal direction of the crystal.

The optical measurements were made with a Shimadzu (Kyoto, Japan) UV3600 UV–VIS-NIR spectrophotometer and an ISR-3100 integrating sphere attachment of 3 mm × 12 mm beam area. All the reflectance measurements were made in the range of 700 to 1,200 nm. The measured sample consists of an approximately 1-cm^2^ colloidal crystal of 360-nm-diameter polystyrene nanospheres electrosprayed onto a glass substrate covered by a 100-nm-thick ITO layer.

## Conclusions

We can conclude that a simple electrospray method is able to produce thick layers of tridimensional order from off-the-shelf colloidal solutions of nanospheres. Polystyrene nanospheres 360 and 780 nm in diameter were electrosprayed onto 1-cm^2^ metalized areas. Experimental work was made to achieve 3D ordered nanostructures up to 20-μm thick in a few minutes, totally avoiding cracks. With the dimensions used in this work, it is shown that the deposited layers behave as a photonic crystal exhibiting a stop band in the NIR, according to theoretical predictions, thereby demonstrating good quality of the deposited layers.

## Competing interests

The authors declare that they have no competing interests.

## Authors’ contributions

AC and SB assembled the electrospray setup and deposited the layers. DH did the spectrometry measurements. LC suggested the use of electrospray for the deposition of colloidal crystals and wrote the paper together with AC and SB. All authors read and approved the final manuscript.
